# Clinical implications and recurrence rates of first-time catheter ablation for atrial fibrillation in hypertrophic cardiomyopathy: a nationwide cohort study

**DOI:** 10.1093/ehjopen/oeag046

**Published:** 2026-03-13

**Authors:** Christopher R Zörner, Lise Da Riis-Vestergaard, Anne-Marie Schjerning, Morten Kvistholm Jensen, Alex Hørby Christensen, Jacob Tfelt-Hansen, Jacob Tønnesen, Charlotte Middelfart, Peter Vibe Rasmussen, Gunnar Gislason, Morten Lock Hansen

**Affiliations:** Department of Cardiology, Herlev-Gentofte University Hospital, University of Copenhagen, Gentofte Hospitalsvej 1, 2900 Hellerup, Denmark; Department of Cardiology, Heart Center, Rigshospitalet, Blegdamsvej 9, 2100 Copenhagen, Denmark; Department of Cardiology, Herlev-Gentofte University Hospital, University of Copenhagen, Gentofte Hospitalsvej 1, 2900 Hellerup, Denmark; Department of Cardiology, Heart Center, Rigshospitalet, Blegdamsvej 9, 2100 Copenhagen, Denmark; The Danish Heart Foundation, Vognmagergade 7, 1120 Copenhagen, Denmark; Department of Cardiology, Aarhus University Hospital, Palle Juul-Jensens Blvd. 99, 8200 Aarhus, Denmark; Department of Cardiology, Herlev-Gentofte University Hospital, University of Copenhagen, Gentofte Hospitalsvej 1, 2900 Hellerup, Denmark; Department of Cardiology, Heart Center, Rigshospitalet, Blegdamsvej 9, 2100 Copenhagen, Denmark; Department of Clinical Medicine, Faculty of Health and Medical Sciences, University of Copenhagen, Blegdamsvej 3B, 2200 Copenhagen, Denmark; Department of Cardiology, Heart Center, Rigshospitalet, Blegdamsvej 9, 2100 Copenhagen, Denmark; Department of Forensic Medicine, University of Copenhagen, Frederik V's Vej 11, 2100 Copenhagen, Denmark; Department of Cardiology, Herlev-Gentofte University Hospital, University of Copenhagen, Gentofte Hospitalsvej 1, 2900 Hellerup, Denmark; Department of Cardiology, Herlev-Gentofte University Hospital, University of Copenhagen, Gentofte Hospitalsvej 1, 2900 Hellerup, Denmark; Department of Cardiology, Herlev-Gentofte University Hospital, University of Copenhagen, Gentofte Hospitalsvej 1, 2900 Hellerup, Denmark; Department of Cardiology, Herlev-Gentofte University Hospital, University of Copenhagen, Gentofte Hospitalsvej 1, 2900 Hellerup, Denmark; The Danish Heart Foundation, Vognmagergade 7, 1120 Copenhagen, Denmark; Department of Clinical Medicine, Faculty of Health and Medical Sciences, University of Copenhagen, Blegdamsvej 3B, 2200 Copenhagen, Denmark; The National Institute of Public Health, University of Southern Denmark, Studiestræde 61455 Copenhagen, Denmark; Department of Cardiology, Heart Center, Rigshospitalet, Blegdamsvej 9, 2100 Copenhagen, Denmark; Department of Clinical Medicine, Faculty of Health and Medical Sciences, University of Copenhagen, Blegdamsvej 3B, 2200 Copenhagen, Denmark

## Abstract

**Aims:**

Catheter ablation (CA) for atrial fibrillation (AF) in patients with hypertrophic cardiomyopathy (HCM) poses unique challenges due to structural and electrical remodelling. This study examines AF recurrence and complication rates following CA in HCM patients compared to a matched AF non-HCM cohort.

**Methods and results:**

A retrospective cohort study of Danish nationwide registries from 2010 to 2022 was conducted. Rates of AF recurrence, heart failure, ischaemic stroke, and death were analysed using propensity score matching and Cox regression models. Among 28 231 patients undergoing first-time CA for AF, 299 had a confirmed diagnosis of HCM. Patients with HCM demonstrated significantly higher rates of AF recurrence compared to a matched non-HCM cohort, with 40% experiencing recurrence vs. 30% in the non-HCM group after 1 year and 67% vs. 58% after 5 years [hazard ratio (HR) 1.41, *P* < 0.001]. Despite the elevated recurrence rates, no significant differences were observed in the incidence of ischaemic stroke (HR 0.99, *P* & 0.9) or all-cause mortality (HR 1.18, *P* & 0.3). However, the risk of heart failure was significantly increased (HR 2.45, *P* & <0.001).

**Conclusion:**

HCM patients experience higher AF recurrence rates post-CA as well as a higher risk of heart failure. These findings highlight the need for tailored strategies to improve outcomes in this high-risk group.

## Introduction

Atrial fibrillation (AF) is a frequently encountered arrhythmia in patients with hypertrophic cardiomyopathy (HCM), a genetic condition characterized by abnormal thickening of the heart muscle, particularly the left ventricle.^1–3^ Remodelling of the heart's architecture, especially within the atria, creates a substrate that predisposes patients to the development of AF.^2,4–6^

The presence of AF in patients with HCM presents significant and distinct challenges for clinicians. For Instance, the symptoms of AF, such as palpitations, fatigue, and shortness of breath, tend to be poorly tolerated in this population.^7,8^

Treating AF in patients with HCM is also more complex compared to the general population. Rate control strategies, such as beta-blockers or non-dihydropyridine calcium channel blockers, are often utilized, but these may have limitations in efficacy and tolerability.^6,9,10^

Another significant concern is the elevated risk of thromboembolic events, including stroke, in patients with both HCM and AF.^11–13^ Therefore, anticoagulation is recommended for all patients with HCM and AF, regardless of their CHA_2_DS_2_-VA score, to mitigate this heightened stroke risk.^14,15^

Given the challenges associated with rate control and the potential for persistent symptoms even with optimal medical therapy, catheter ablation (CA) has emerged as a viable treatment option for rhythm control in AF.^16^ This procedure has gained prominence as an effective strategy to restore and maintain sinus rhythm, thereby potentially improving symptoms and quality of life in HCM patients with AF.^17^

However, the effectiveness of CA in patients with HCM appears to be lower than in other patient populations. Studies suggest that patients with HCM undergoing CA for AF have higher rates of AF recurrence post-procedure.^18–22^ These findings highlight the need for careful patient selection and counselling regarding the expected outcomes of CA in this patient group.

Despite its potential, there is a paucity of robust data regarding the outcomes of CA in HCM patients with AF. Existing studies are limited by small sample sizes and short follow-up periods, leaving many questions unanswered. To address this gap, the present study leverages nationwide registries in Denmark to compare the outcomes of CA in HCM patients with a matched cohort. This study aims to provide valuable insights into the efficacy and safety of CA in this unique patient population.

## Methods

### Study design and population

This study was designed as a retrospective observational cohort study. All patients with registered AF who underwent CA in Denmark between 1 January 2010 and 31 December 2022 were included at the time of CA procedure. Patients with a HCM diagnosis were compared to a matched cohort of patients without HCM.

### Outcomes of interest

The primary outcome was recurrence of AF following CA. AF recurrence was defined as a composite endpoint comprising the first occurrence of new AF admission, redemption of antiarrhythmic drug (AAD) prescription (including Amiodarone and class 1C ADDs), cardioversion, and re-ablation. This approach has been utilized in published studies examining AF recurrence in the Danish registries.^23–25^ As a secondary endpoint, incidence of heart failure, ischaemic stroke, and death was compared between groups.

### Data sources

This study was based on several nationwide registers: The Danish National Patient Register,^26^ The Civil Registration System,^27^ The Danish Registry of Medicinal Product Statistics as well as the Danish National Prescription Registry.^28^ These nationwide registers were cross-linked on the individual level using the unique permanent identification number given to all Danish residents at birth or migration. Primary diagnosis is registered according to the International Classification of Diseases; the 10th revision (ICD-10) since 1994. Procedures are coded according to the Nordic Classification of Surgical Procedures (NCSP) by The Nordic Medico-Statistical Committee. These registries have been utilized for cardiovascular research in numerous published studies and the methodology is well established.^29,30^

### Variables

Comorbidities described at baseline included heart failure, ischaemic heart disease (IHD), ischaemic stroke, chronic kidney disease (CKD), hypertension, and chronic obstructive pulmonary disease (COPD), and were registered up to five years before study inclusion. ICD-10 codes used for these comorbidities are provided in the Supplementary material online, table  *S1*.

Concomitant pharmacotherapy at baseline was defined as any claimed prescription 180 days before the date of study inclusion. Medications included in the analysis were beta-blockers, calcium channel blockers (CCB), ACE inhibitors, loop diuretics, spironolactone, oral anticoagulant therapy (OAC), digoxin, and amiodarone. ATC codes used for these pharmaceuticals are provided in Supplementary material online, *Table S2*.

### Matching

Analysis was conducted between HCM patients undergoing CA for AF and a matched cohort of HCM-free patients undergoing the same procedure. Matching was achieved via 1:4 propensity score matching, adjusting for patient age, sex, as well as comorbidities including hypertension, IHD, CKD, and COPD. Nearest neighbour matching without replacement was employed to avoid duplication of control subjects. A calliper of 0.2 standard deviations of the logit of the propensity score was used to ensure close matching. All patients with HCM were matched to controls where possible. Propensity score matching has been increasingly utilized as a reliable method in register-based studies.

### Statistical analysis

Descriptive tables were used to depict the study population by morbidity burden, with continuous variables reported as medians and interquartile ranges [IQRs] and categorical variables summarized with counts and corresponding percentages. The cumulative incidence of AF recurrence was calculated utilizing the Aalen–Johansen estimator and death accounted for as a competing risk factor. Cox proportional-hazard analysis was used to examine the association between HCM status and stroke, heart failure, and death, with estimates reported as hazard ratios (HR) with 95% confidence intervals (95% CI)

Statistical analysis and data management were conducted using R statistical software [R Core Team (2021). R: A language and environment for statistical computing. R Foundation for Statistical Computing, Vienna, Austria. URL https://www.R-project.org/.]

### Ethics

According to Danish legislation, retrospective studies using administrative health databases do not require ethical approval in Denmark. The Danish Data Protection Agency has approved the use of registry data, and the current project is registered (Approval number: *P*-2019-408).

### Data availability

Data for this study are derived from and accessed through Statistics Denmark. By law, these data are not allowed to be shared. For this reason, data cannot be made available to other researchers.

## Results

### Demographics

A total of 28 231 patients with a recorded history of AF underwent first-time CA for AF between 2010 and 2022. Of these 299 had a registered diagnosis of HCM. Comparing the group of patients with an HCM history to those without revealed that at time of procedure patients in the HCM groups were generally younger, with a median age of 61 years (IQR 55–68) compared to 63 years (IQR 56–69) in the general AF population. Sex distribution showed male predominance in both groups, with 31% of patients being female in the HCM group vs. 28% in the general group. Prevalence of cardiovascular comorbidity was generally high, though the patients with HCM presented with significantly higher rates of comorbidity than those without. This was especially the case for prevalence of IHD (25% vs. 15%), hypertension (58% vs. 47%) and heart failure (26% vs. 17%).

Finally, in terms of concomitant pharmacotherapy there were notable differences among the cohort, with HCM patients more frequently prescribed loop diuretics (28% vs. 16%), spironolactone (16% vs. 7%), RAS inhibitors (39% vs. 45%) and amiodarone (25% vs. 18%). Other drugs analysed were prescribed evenly between the populations. (*Table 1*)

**Table 1:** Baseline characteristics: all patients undergoing catheter ablation with and without HCM

	Without HCM	With HCM	*P* - value
Number of patients	27932	299	
Age: median in years [median (IQR)]	63.60 [56.10, 69.90]	61.90 [55.00, 68.70]	0.04
Sex: Female, *n*, (%)	7858 (28.1)	94 (31.4)	0.23
Ischaemic heart disease (%)	4186 (15.0)	75 (25.1)	<0.001
Chronic obstructive pulmonary disease (%)	1054 (3.8)	17 (5.7)	0.12
Chronic kidney disease (%)	614 (2.2)	13 (4.3)	0.02
Hypertension	12981 (46.5)	172 (57.5)	<0.001
Heart failure (%)	4644 (16.6)	79 (26.4)	<0.001
Ischaemic stroke (%)	1438 (5.1)	21 (7.0)	0.19
Thiazide diuretics (%)	2498 (8.9)	21 (7.0)	0.29
Spironolactone (%)	2073 (7.4)	48 (16.1)	<0.001
Loop diuretics (%)	4564 (16.3)	85 (28.4)	<0.001
Beta blockers (%)	18650 (66.8)	208 (69.6)	0.34
Calcium channel blockers, all (%)	5415 (19.4)	61 (20.4)	0.71
Digoxin (%)	3836 (13.7)	39 (13.0)	0.80
Amiodarone (%)	4999 (17.9)	75 (25.1)	0.00
Verapamil (%)	1963 (7.0)	27 (9.0)	0.22
RAS inhobitors (%)	10777 (38.6)	133 (44.5)	0.04
ACE inhibitors (%)	6175 (22.1)	74 (24.7)	0.31
Angiotensin II converting enzyme inhibitors	4974 (17.8)	67 (22.4)	0.49
Oral anticoagulant therapy, all (%)	23770 (85.1)	258 (86.3)	0.62
Warfarin (%)	12127 (43.4)	136 (45.5)	0.51
Phenprocoumon (%)	131 (0.5)	4 (1.3)	0.08
Edoxaban (%)	615 (2.2)	6 (2.0)	0.98
Apixaban (%)	5137 (18.4)	57 (19.1)	0.82
Rivaroxaban (%)	5384 (19.3)	56 (18.7)	0.87
Dabigatran (%)	3516 (12.6)	25 (8.4)	0.04

For further analysis, patients with HCM were compared to a matched cohort. Patient characteristics, comorbidities and pharmacotherapy were evenly matched between the two cohorts with no significant differences detected. (*Table 2*)

**Table 2:** Baseline characteristics: patients undergoing catheter ablation with HCM vs. a matched cohort

	Without HCM	With HCM	*P*-value
Number of patients	1196	299	
Age: median in years [median (IQR)]	62.65 [55.50, 69.10]	61.90 [55.00, 68.70]	0.57
Sex: Female, *n*, (%)	370 (30.9)	94 (31.4)	0.92
Ischaemic heart disease (%)	308 (25.8)	75 (25.1)	0.87
Chronic obstructive pulmonary disease (%)	45 (3.8)	17 (5.7)	0.18
Chronic kidney disease (%)	66 (5.5)	13 (4.3)	0.51
Hypertension	712 (59.5)	172 (57.5)	0.57
Heart failure (%)	325 (27.2)	79 (26.4)	0.85
Ischaemic stroke (%)	62 (5.2)	21 (7.0)	0.27
Thiazide diuretics (%)	140 (11.7)	21 (7.0)	0.03
Spironolactone (%)	130 (10.9)	48 (16.1)	0.02
Loop diuretics (%)	253 (21.2)	85 (28.4)	0.01
Beta blockers (%)	861 (72.0)	208 (69.6)	0.45
Calcium channel blockers, all (%)	249 (20.8)	61 (20.4)	0.94
Digoxin (%)	199 (16.6)	39 (13.0)	0.15
Amiodarone (%)	253 (21.2)	75 (25.1)	0.16
Verapamil (%)	77 (6.4)	27 (9.0)	0.15
RAS inhobitors (%)	583 (48.7)	133 (44.5)	0.21
ACE inhibitors (%)	331 (27.7)	74 (24.7)	0.34
Angiotensin II converting enzyme inhibitors	269 (22.5)	67 (22.4)	1.00
Oral anticoagulant therapy, all (%)	1041 (87.0)	258 (86.3)	0.80
Warfarin (%)	542 (45.3)	136 (45.5)	1.00
Phenprocoumon (%)	4 (0.3)	4 (1.3)	0.09
Edoxaban (%)	25 (2.1)	6 (2.0)	1.00
Apixaban (%)	229 (19.1)	57 (19.1)	1.00
Rivaroxaban (%)	224 (18.7)	56 (18.7)	1.00
Dabigatran (%)	148 (12.4)	25 (8.4)	0.07

### Cumulative incidence of AF recurrence

**Figure 1:**
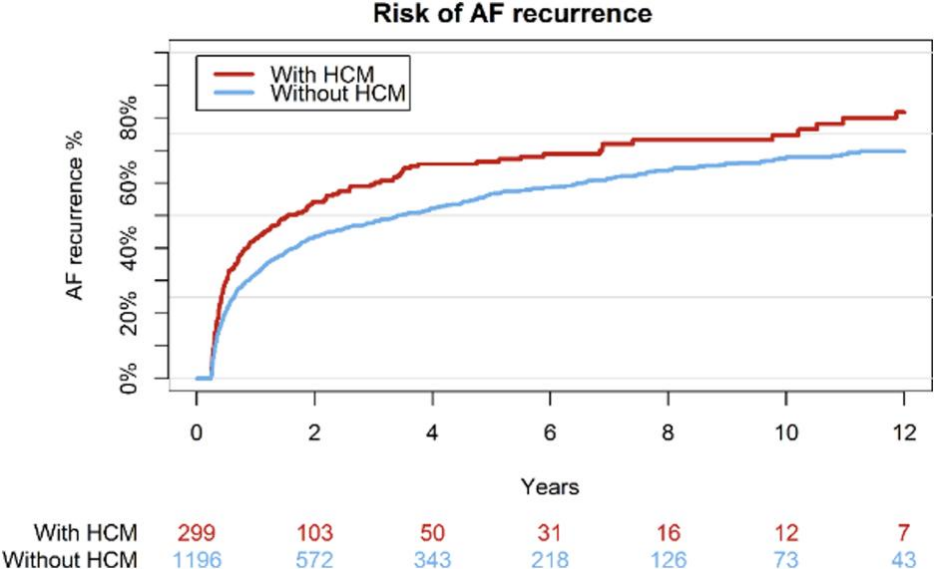
Cumulative incidence of AF recurrence.

**Figure 2:**
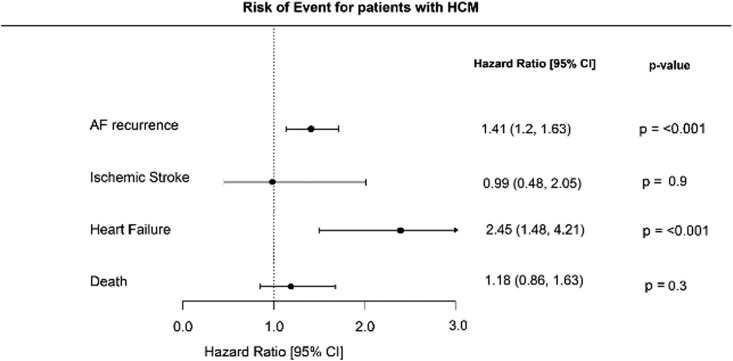
Forest plot: risk of AF recurrence, ischaemic stroke, heart failure, and death.

One year after CA, 40% of AF patients with HCM were registered with recurrence of AF while this was the case for 30% of patients in the matched cohort. After 5 years, this disparity increased to 67% AF recurrence rate for the HCM group, while recurrence was registered at 58% in the control group. Over the full observation period of 12 years 82% for patients with HCM and 71% of patients without experienced AF recurrence. This discrepancy was calculated to be significant with a *P* value of <0.001 and HR of 1.56 (95% CI 1.2–2.0). These findings are illustrated in *Figure 1* and *Figure 2*. Analysing the distribution of first registered events among HCM and non HCM patients revealed a comparable distribution of patients who received DC conversion (7.4% vs. 8.9%) and admission for AF (43.9% vs. 39.4%). (*Table 3*)

**Table 3:** Characteristics of patients with HCM: with and without AF-recurrence

	Without HCM	With HCM
Re-ablation, *n*, (%)	<4	4 (2.2)
DC conversion, *n*, (%)	16 (8.9)	13 (7.2)
AF admission, *n*, (%)	71 (39.4)	79 (43.9)
Amiodarone, *n*, (%)	24 (13.3)	34 (18.9)
Class 1 C AADs, *n*, (%)	6 (3.3)	9 (5)

### Risk of ischaemic stroke, heart failure, and death

While there was a significantly increased risk of AF recurrence in patients with HCM, there was also a significantly increased risk of cardiovascular complications among these patients compared to the matched cohort. Risk of developing heart failure was determined at HR 2.45 (95% CI 1.48–4.21, *P*-value & < 0.001) for patients with HCM, while risk of ischaemic stroke was calculated to be equal between the groups (HR 0.99, 95% CI 0.48–2.05, *P*-value & 0.9). Finally, the risk of death for patients with HCM was calculated to HR 1.18 (95% CI 0.86–1.63, *P*-value & 0.3) These findings are summarized in *Figure 2*.

## Discussion

Patients with HCM who underwent first-time CA for AF experienced significantly higher rates of AF recurrence compared to a matched non-HCM cohort. In addition to this, HCM was also associated with an increased risk of developing heart failure, while there was no significant difference in the risk of ischaemic stroke or death between the HCM and non-HCM cohorts. These findings highlight the challenges of achieving long-term rhythm control in HCM patients. The structural abnormalities inherent to HCM, such as myocardial fibrosis and atrial remodelling, likely contribute to the reduced efficacy of CA as well as to the high symptomatic burden HCM patients with AF experience.^4,31^

This study's findings add to the corpus of evidence from previous research investigating CA in a contemporary HCM population. Published studies on the topic predominantly support the notion of increased AF recurrence rates in HCM patients compared to the general population.

Most comparable studies are vastly limited by low sample size, ranging between 14–40, with a single Chinese study including 97 patients with HCM.^32–37^ Furthermore in previous studies ablation techniques, burden of AF and patient characteristics are not uniformly registered and defined, making comparison of their outcome challenging. Despite these limitations, the existing evidence highlights the challenges, complexities and potential benefits of CA in this complex and multifaceted patient group.^19^

The increased recurrence suggests that, while CA remains a viable treatment option, it may be less effective in achieving long-term rhythm control in this population. As a result, clinicians should carefully consider patient selection for CA, balancing the potential benefits with the high likelihood of recurrence. For patients undergoing CA, there may be a need for more aggressive post-ablation monitoring, including the use of antiarrhythmic medications and potential re-ablation procedures.^31^ Additionally, these results underscore the importance of managing patient expectations regarding CA outcomes. HCM patients should be informed about the increased risk of AF recurrence and the potential for ongoing management even after ablation. Future research should focus on optimizing CA techniques for HCM patients, perhaps through the use of advanced imaging to better target fibrotic areas or exploring new technologies to improve ablation efficacy.

Despite the higher recurrence rates, our study did not demonstrate a significantly increased risk of ischaemic stroke or death in patients with HCM. This observation could reflect effective post-procedural management, including anticoagulation therapy, which is routinely recommended for all HCM patients with AF, regardless of traditional stroke risk scores. However, we did detect a significantly increased risk of heart failure development in the HCM group. This finding suggests that monitoring for early signs of heart failure should be prioritized post-procedure and during follow-up.

### Strengths and limitations

Strengths of this study include the use of nationwide registry data, facilitating the option of a long follow-up period and a large sample size. Given the relative rarity of HCM, finding a suitable number of patients for prospective trials is challenging, making the utilization of observational data well suited for studies of this patient group.^38^

Several limitations of our study must be acknowledged. First, the retrospective nature of the study and reliance on registry data may introduce selection and information bias. While propensity score matching was employed to balance baseline characteristics between HCM and non-HCM patients, unmeasured confounders may still influence the outcomes.

While the Danish nationwide registries provide access to a wide range of data points and observations, certain measurements, scores, and values, important for the accurate classification and contextualization of both patients with HCM and those undergoing CA patients are absent.^39^ Most notably, the utilized databases do not contain measurements and values from echocardiography, such as left ventricular ejection fraction or LVOTO and severity of symptoms. The same is the case for the results of genetic testing. With the absence of such values, the diagnosis of HCM in this study relies on registered ICD-10 codes and the judgement of the treating physician. Given that HCM can be challenging to diagnose and confounded with conditions that likewise present with left ventricular hypertrophy, misclassification and error cannot be excluded. A large Danish validation study examining the accuracy of diagnosis within the Danish registries reported the ICD-10 code for HCM to have a 90% positive predictive value when used; however, the sample size was comparatively low.^40^

Due to the lack of differentiation in the registries between various subtypes of AF, such as paroxysmal, persistent, and permanent AF, the influence of AF type on CA outcome could not be evaluated. Furthermore, patients’ EHRA score, which classifies the severity of AF related symptoms, is not registered in these registries.

Lastly, while this study examines the outcome of more HCM patients undergoing CA than any other previous study to our knowledge, a larger sample size would be ideally needed to accurately determine the risk of complications in this patient group. Large variations described in 95% CI exemplify this problem.

## Conclusion

In summary, while CA remains a key therapeutic option for AF in HCM, its limitations necessitate a more nuanced and personalized approach to treatment. Ongoing research and clinical innovation are essential to improving outcomes for these high-risk patients.

## Supplementary Material

oeag046_Supplementary_Data
