# Supplementary material for: Clinical implications and recurrence rates of first-time catheter ablation for atrial fibrillation in hypertrophic cardiomyopathy: a nationwide cohort study
Source: Eur Heart J Open. 2026 Mar 13;6(2):oeag046. doi: 10.1093/ehjopen/oeag046 (PMC13021360; doi:10.1093/ehjopen/oeag046)
Supplement: oeag046_Supplementary_Data [file oeag046_supplementary_data.docx]

## **Supplementary table 1**

Diagnostic and procedural codes used

| Catheter ablation | BFF03, BFF04 |
| --- | --- |
| Hypertrophic cardiomyopathy | ICD-10: DI421, DI422 |
| Atrial fibrillation / Atrial flutter | ICD-10: I48 |
| Chronic Obstructive Pulmonary Disease | ICD-10: J42-J44 |
| Ischemic heart disease | ICD-10: DI20, DI21, DI22, DI23, DI24, DI252, DI255, DI256,  DI258, DI259, DT822,  DZ951I25 |
| Hypertension | Treatment with more than one anti-hypertensive medication. |
| Congestive heart failure | ICD-10: I50 |
| Ischemic stroke / TIA / systemic embolism | ICD-10: I63, I64, I74, G458, G459 |
| Chronic Kidney Disease | ICD-10: N02-N08, N11-N14, N18, N19, N26, N158-N160, N 162, N163, N164, N168, Q61, E102, E112, E132, E142, I120, M321B |

##

## **Supplementary table 2**

ATC-codes used to define medical therapy

| Oral anticoagulant treatment  (Warfarin, phenprocoumon, dabigatran, rivaroxaban, apixaban, edoxaban) | ATC-codes:  B01AA03, B01AA04, B01AE07, B01AF01, B01AF02, B01AF03 |
| --- | --- |
| Beta-blockers | C07A, C07B, C07C, C07D, C07F |
| Calcium channel antagonist | C08C, C08D, C08E, C08G, C09BB, CO9DB |
| Spironolactone | C03D, C03E, C03EB |
| Loop diuretics | C03C, C03EB |
| Non-loop diuretics | C02L, C02DA, C07D, C09XA52, C03A, C03EA, C03B, C03X, C07C, C08G, C09BA, C09DA, C03D, C03E, C03EB |
| ACE inhibitors | C09A, C09B |
| Digoxin | C01AA |
| Amiodarone | C02BD01 |
